# Relationship between health-related determinants and adherence to breast and colorectal cancer screening: a population-based study in Flanders, Belgium

**DOI:** 10.1093/eurpub/ckad206

**Published:** 2023-11-24

**Authors:** Allegra Ferrari, Thuy Ngan Tran, Sarah Hoeck, Marc Peeters, Mathijs Goossens, Guido Van Hal

**Affiliations:** Social Epidemiology and Health Policy, Family Medicine and Population Health (FAMPOP), University of Antwerp, Antwerp, Belgium; Department of Health Sciences (DISSAL), University of Genoa, Genoa, Italy; Social Epidemiology and Health Policy, Family Medicine and Population Health (FAMPOP), University of Antwerp, Antwerp, Belgium; Centre for Cancer Detection, Bruges, Belgium; Social Epidemiology and Health Policy, Family Medicine and Population Health (FAMPOP), University of Antwerp, Antwerp, Belgium; Centre for Cancer Detection, Bruges, Belgium; Department of Oncology, Antwerp University Hospital, Antwerp, Belgium; Integrated Personalized & Precision Oncology Network (IPPON), University of Antwerp, Antwerp, Belgium; Centre for Cancer Detection, Bruges, Belgium; The Vrije Universiteit Brussel (VUB), Brussels, Belgium; Social Epidemiology and Health Policy, Family Medicine and Population Health (FAMPOP), University of Antwerp, Antwerp, Belgium; Centre for Cancer Detection, Bruges, Belgium

## Abstract

**Background:**

Despite the recognized benefits of structured cancer screening, tests outside organized screening programs are common. Comprehensive reports on outside program screening in Europe are lacking, but the Flemish breast cancer (BC) and colorectal cancer (CRC) screening programs monitor data on non-organized tests prescribed by GPs and specialists.

**Methods:**

Using data at aggregated level, logistic regression was used to examine the relationship between health care utilization and screening coverage in 308 Flemish municipalities during 2015–18.

**Results:**

With regards to BC, municipalities with higher rates of gynecologists’ visits had lower odds of coverage inside (−8%) and higher odds of coverage outside (+17%) the program. By contrast, municipalities with higher rates of GP visits, had higher odds of coverage inside (+6%) and lower odds of coverage outside (−7%) the program. As for CRC, municipalities with higher rates of visits gastroenterologists’ visits had lower odds of coverage inside (−3%). Instead, municipalities with higher rates of GP visits, had higher odds of coverage both inside (+2%) and outside (+5%) the program. Municipalities with higher percentages of people with chronic conditions had higher odds of coverage within both the BC and CRC programs (+5% and +3%), and lower odds of outside screening (−7% and −6%). Municipalities with higher percentages of people 65+ with dementia and with mood disorders had, respectively, higher odds (+13% and +5%) and lower odds (−3% and −4%) of coverage inside both the BC and CRC programs.

**Conclusion:**

Our findings underscore the impact of healthcare utilization on cancer screening coverage at the municipal level in Flanders.

## Introduction

Breast cancer (BC) is the most common cancer worldwide, with over two million cases each year.^1^ In 2019 it was the main cause of cancer-related disability-adjusted life years (DALYs) and cancer-related deaths among females worldwide.^2^ Belgium has the highest global rate of BC,^1^ and the highest age-standardized incidence rate is found in Flanders, its most populous region (103.4 per 100 000 person-years in 2020).^3^ Colorectal cancer (CRC) is the third most commonly diagnosed cancer in males and the second in females worldwide.^1^ For both sexes, in 2019 it was the second cause of cancer-related DALYs and third cause of cancer death.^2^ With Belgium ranking 14th in terms of incidence rate,^4^ in Flanders in 2020 CRC was the fourth most common cancer (22.8 and 31.9 per 100 000 person-years, in females and males).^3^

The early diagnosis of BC and CRC through screening increases the chances of a favorable prognosis. For the lifetime of single-year age cohorts in the US (data year 2016), screening can prevent over 10 000 deaths from BC and 74 000 deaths from CRC among 50-year-old women and 50-year-old men and women, respectively.^5^ Likewise, if effective BC and CRC screening programs were in place in all European countries additional 12 500 and 80 000 cancer deaths could be prevented each year.^6^^,^^7^

Even though screening has been proven to be beneficial, participation in Flanders is still below the levels recommended by the European Commission,^8^^,^^9^ with about 15% and 25% of the population from the target groups never screened for BC and CRC, respectively.^10^

One of the most powerful independent predictors of a person's choice to have a cancer screening test is a doctor's advice.^11^ Previous evidence has shown that health status, healthcare access and healthcare utilization are associated with participation in both the BC and CRC screening program in Flanders.^12–14^ Similar observations have been made over time by researchers using various study designs.^15–19^ Only a few of these reports, however, are recent and none of them take in account the differential impact that these determinants may have on screening coverage inside (organized) and outside (opportunistic or non-organized) the official cancer screening program.

Testing outside of the organized program occurs when a patient asks a healthcare provider for a test or when the healthcare provider, often because there is a sign, symptom, or medical condition that leads to the recommendation, offers the test.

Organized screening programs recruit large numbers of people who are offered the same services, information and support. Because organized screening programs have to be of a high standard, the screening services are checked and monitored by independent bodies. Certain shortcomings, e.g. a lack of quality control by health authorities, disparities in screening access based on one’s perception of risk or willingness and ability to pay, etc. can so only be avoided through the dissemination of a structured organized program.^20^

While comprehensive reports on opportunistic screening in Europe are lacking, the Flemish BC and CRC screening programs also monitor data on tests prescribed outside of the organized screening program.

Health-related determinants may have a differential impact on coverage inside and outside the organized screening program. Therefore, this study aimed to assess the associations between several health-related determinants and coverage inside and outside the organized screening programs for BC and CRC.

## Methods

### Study design and data source

Municipality-level health-related data were linked to data on coverage inside and outside the organized screening programs for BC and CRC among respective target populations in 308 Flemish municipalities, between 2015 and 2018.

Data on screening coverage in Flanders was retrieved from the Centre for Cancer Detection (CvKO) (https://www.bevolkingsonderzoek.be; accessed 30 October 2022). The Belgian InterMutualist Agency (IMA) databank was used to obtain information on healthcare utilization (http://atlas.ima-aim.be/databanken; accessed 30 October 2022). Finally, the Flemish provincial authorities' databank was accessed for data on sociodemographic characteristics (https://provincies.incijfers.be/databank; accessed 30 October 2022).

### Study setting

A BC screening program has been in place in Flanders since 2001.^21^ Eligible women aged 50–69 are recruited for the program through a personalized invitation letter with a set time and location. A mammography is provided every two years and is paid for by the health insurance system.

A CRC screening program has been in place in Flanders since 2013.^22^ Target ages have been gradually extended from 56–74 in 2013 to 50–74 in 2020. Men and women aged 50–74 are recruited for the program every two years. The invitation contains a free fecal immunochemical test (FIT) that can be administered at home and then sent to the central lab with the included envelope.

Both programs align with the European Guidelines for Quality Assurance.^8^^,^^9^

### Statistical analysis

Logistic regression for aggregated data was used to evaluate the associations between health-related determinants and screening coverage. In particular, four distinct analyses were conducted to study the association between health-related determinants and coverage: (i) inside the organized BC screening program; (ii) outside the organized BC screening program; (iii) inside the organized CRC screening program; and (iv) outside the organized CRC screening program.

Per each municipality, a minimum of six and two individuals were covered inside of the program and a minimum of eight and one individuals were covered outside of the program for BC and CRC screening, respectively.

Because, as a rule of thumb, at least 10 outcome events per determinant are required for accurate coefficient estimation in logistic regression models,^23^ and a maximum of 15 variables were used for each model, our sample size could provide sufficient statistical power.

To account for the repeated measurements across the years, a Generalized Estimating Equations model with AutoRegressive correlation structure was used.

Since only aggregated data at the municipal level were used, and data for cells with less than five events were not included, privacy was maintained. As missing data was minimal (<3%) and solely due to privacy concerns, records with missing data were removed from the dataset.

Descriptive statistics were reported as median and range. In the logistic regression model, adjusted odds ratios (aORs) were reported with 95% confidence intervals (95% CIs). Multicollinearity in multivariate models was checked using variance inflation factors (VIFs).

The Benjamini–Hochberg Procedure^24^ was used to control for multiple testing and minimize the possibility of false positives. Any *P* values less than the adjusted critical threshold based on a 0.05 false discovery rate was considered statistically significant. Conversely, *P*-values exceeding this threshold were considered nonsignificant. Multiple testing correction is shown in Supplementary table S6. All analyses were performed with R (version 4.0.3).

### Outcomes

With regards to BC, inside coverage (for a given year) was measured as the number of females who completed a mammography inside the organized program out of the total number of females at target ages in previous 2 years.

Outside coverage (for a given year) was measured as the number of females who had a mammography outside the organized program (prescribed by GPs/specialists) out of the total number of individuals at target ages in previous 2 years.^25^

For CRC, inside coverage (for a given year) was measured as the number of individuals who completed a fecal occult blood test (FOBT) inside the organized program out of the total number of individuals at target ages in previous 2 years.

Outside coverage (for a given year) was measured as the number of individuals who completed an FOBT outside the organized program out of the total number of individuals at target ages in previous 2 years.^26^

### Determinants and covariates for adjustment

Eleven health-related parameters were investigated as potential factors associated with screening coverage. A full description of the main determinants of assessment is provided in table 1.

**Table 1:** Health-related determinants of assessment

Determinants	Description
GP visits (%)	Percentage of residents with health insurance with at least 1 GP contact (consultation/visit) in the past year
Visits with gynecologist (%)	Percentage of residents with health insurance with at least one consultation with a gynecologist in the past year
Visits with gastroenterologist (%)	Percentage of residents with health insurance with at least one consultation with a gastroenterologist in the past year
Preventive dental visits (%)	Share of residents with health insurance with at least 2 preventive contacts with the dentist in 2 different years within a period of 3 years
Chronic conditions (%)	Percentage of residents with health insurance with at least one chronic condition status (compared with persons in health insurance) per calendar year
Diabetes (‰)	Persons with episodes of antidiabetic drugs or with a nomenclature referring to diabetes (diabetes convention, diabetes pass, diabetes care program), per 1000 residents with health insurance per calendar year
Disabilities (‰)	Persons with disabilities, recognized by the Directorate-General for Disabled Persons, per 1000 people over the age of 18 per calendar year
Dementia (%)	Percentage of residents with health insurance of at least 65 years who use antidementia per calendar year
Mood disorders (%)	Percentage of antidepressants users per calendar year
Psychotic disorders (%)	Percentage of antipsychotic users per calendar year
Alcohol addiction (%)	Percentage of drug users for alcohol dependence per calendar year

Users of antidementia drugs, antidepressants, antipsychotic drugs and anti-alcohol addiction drugs were used as proxy measures for, respectively, people with dementia, mood disorders, psychotic disorders and with alcohol addiction.

Twelve socio-demographic variables (sex, age, position in the labor market, educational level, average income, residential stability, nationality, having children, having a partner) were used as covariate for adjustment in the multivariate model. Additional details are reported online (https://provincies.incijfers.be/databank).

To minimize possible collider biases,^27^ a causal directed acyclic graph (DAG) based on prior knowledge about the Flemish organized screening programs^12–14^ was used to identify covariates for adjustment.

To conceptualize the exposure-outcome relationship, a multidisciplinary brainstorming session was organized among a medical doctor, three epidemiologists (among whom the program managers of the Flemish screening programs) and a sociologist. DAGs are well-established methods for the analysis of causal inference in epidemiology and are used to show how associations translate into causal relations.^28^

The DAGs and the selected covariates for adjustment are available in Supplementary figures S1 and S2 and Supplementary tables S1 and S2.

## Results

### Municipal characteristics

Data from all 308 municipalities in Flanders for the period between 2015 and 2018 were included.

Considering BC screening, the overall median coverage was 51.20% (44.40–55.70%) inside the organized program and 11.90% (8.60–17.40%) outside the organized program. For CRC the overall median coverage was 37.70% (34.78–40.60%) inside the organized program and 3.60% (2.80–4.90%) outside the organized program. Median values and 25th–75th percentiles of BC and CRC screening for each year are available in Supplementary table S3. Median values and 25th–75th percentiles of the included municipal characteristics are shown in table 2.

**Table 2:** Characteristics of 308 municipalities during the study period (2015–18); median (25th–75th percentiles)

Municipal characteristics	2015	2016	2017	2018
Covariates for adjustment				
Sex (F) (%)	50.50 (0.89)	50.40 (1.00)	50.40 (0.92)	50.50 (1.00)
Age	43.00 (2.00)	43.00 (2.00)	44.00 (3.00)	44.00 (3.00)
Jobseekers (%)	1.80 (0.70)	1.70 (0.70)	1.60 (0.60)	1.40 (0.60)
Wage earners (%)	36.55 (3.30)	36.60 (3.40)	36.80 (3.40)	37.10 (3.40)
Self-employed (%)	7.90 (2.40)	08.00 (2.32)	8.05 (2.42)	8.20 (2.50)
Retired (%)	19.60 (2.32)	19.90 (2.30)	20.10 (2.30)	20.25 (2.30)
Students in higher education (%)	44.30 (10.02)	44.55 (9.57)	45.20 (10.65)	46.50 (10.22)
Average income (EUR)	18977.50 (2873.25)	18888.00 (3067.50)	19540.00 (3018.50)	19436.33 (3203.00)
Same address as previous year (%)	92.30 (1.70)	92.50 (2.00)	92.35 (1.80)	92.30 (1.80)
Foreign nationality (non-Belgian/Dutch) (%)	2.60 (2.30)	3.00 (2.50)	3.30 (2.52)	3.45 (2.52)
With children (%)	30.30 (1.62)	30.10 (1.70)	29.95 (1.62)	29.80 (1.65)
With a partner (%)	52.70 (2.92)	52.60 (2.72)	52.60 (2.8)	52.55 (2.75)
Health-related variables				
GP visits (%)	84.20 (3.92)	84.00 (3.89)	84.40 (4.12)	86.00 (4.00)
Visits with gynecologist (%)	12.49 (2.30)	12.20 (2.30)	12.20 (2.20)	12.10 (2.40)
Visits with gastroenterologist (%)	4.70 (1.05)	4.60 (0.10)	4.80 (1.10)	4.60 (1.10)
Preventive dental visits (%)	34.60 (6.82)	37.35 (7.12)	40.00 (7.40)	40.65 (6.95)
Chronic conditions (%)	9.70 (1.82)	10.40 (1.82)	11.00 (2.02)	11.50 (2.10)
Diabetes (‰)	52.00 (9.00)	53.00 (9.00)	54.00 (9.00)	55.00 (9.00)
Disabilities (‰)	64.65 (28.61)	65.12 (28.08)	64.44 (28.41)	64.79 (29.80)
Dementia (%)	1.80 (0.80)	1.70 (0.70)	1.70 (0.80)	1.60 (0.70)
Mood disorders (%)	12.10 (2.02)	12.00 (2.12)	12.00 (2.12)	12.05 (2.10)
Psychotic disorders (%)	3.40 (1.00)	3.40 (1.05)	3.30 (1.10)	3.30 (1.00)
Alcohol addiction (%)	0.20 (0.08)	0.20 (0.09)	0.20 (0.08)	0.21 (0.08)

### Association between health-related variables and BC screening

With respect to contact with healthcare providers, municipalities with a higher rate of visits with gynecologists had 8% lower odds of mammography inside of the organized program [aOR 0.92 (95% CI 0.91–0.93); *P* < 0.0001] and 17% higher odds of a mammography outside of the program [aOR 1.17 (95% CI 1.15–1.19); *P* < 0.0001]. By contrast, municipalities with a higher rate of GP visits had 6% higher odds of mammography inside of the organized program [aOR 1.06 (95% CI 1.05–1.07); *P* < 0.0001] and 7% lower odds of mammography outside the program [aOR 0.93 (95% CI 0.92–0.94); *P* < 0.0001].

As for health status, municipalities with a higher percentage of people with chronic conditions had 5% more odds of mammography inside the BC screening program [aOR 1.05 (95% CI 1.03–1.07); *P* < 0.0001] and 7% odds of mammography outside of the program [aOR 0.93 (95% CI 0.90–0.96); *P* < 0.0001]. In addition, municipalities with a higher percentage of people with diabetes and disabilities had 2% lower odds of coverage by opportunistic mammography outside of the organized program [aOR 0.98 (95% CI 0.987–0.988); *P* < 0.0001 and 0.97 (95% CI 0.982–0.985); *P* < 0.0001, respectively].

Municipalities with a higher percentage of people with dementia had 13% higher odds of mammography inside the organized BC screening program [aOR 1.13 (95% CI 1.08–1.17); *P* < 0.0001], and 14% lower odds outside of the organized program [aOR 0.86 (95% CI 0.83–0.91); *P* < 0.0001].

Municipalities with a higher percentage of people with mood and psychotic disorders had 3% lower odds of mammography in the organized BC screening program [aOR 0.97 (95% CI 0.95–0.99); *P* = 0.0004 and 0.97 (95% CI 0.94–0.99); *P* = 0.0166, respectively]. By contrast, with regards to mammography outside the organized program, municipalities with a higher percentage of people with psychotic disorders showed 12% higher odds of coverage [aOR 1.12 (95% CI 1.07–1.17); *P* < 0.0001].

Finally, although with a large confidence interval, municipalities with a higher percentage of people with alcohol addiction showed 65% higher odds of opportunistic mammography [aOR 1.65 (95% CI 1.12–2.44); *P* = 0.0119].

The rate of preventive dentals visits did not have any significant impact on BC screening coverage.

**Figure 1:**
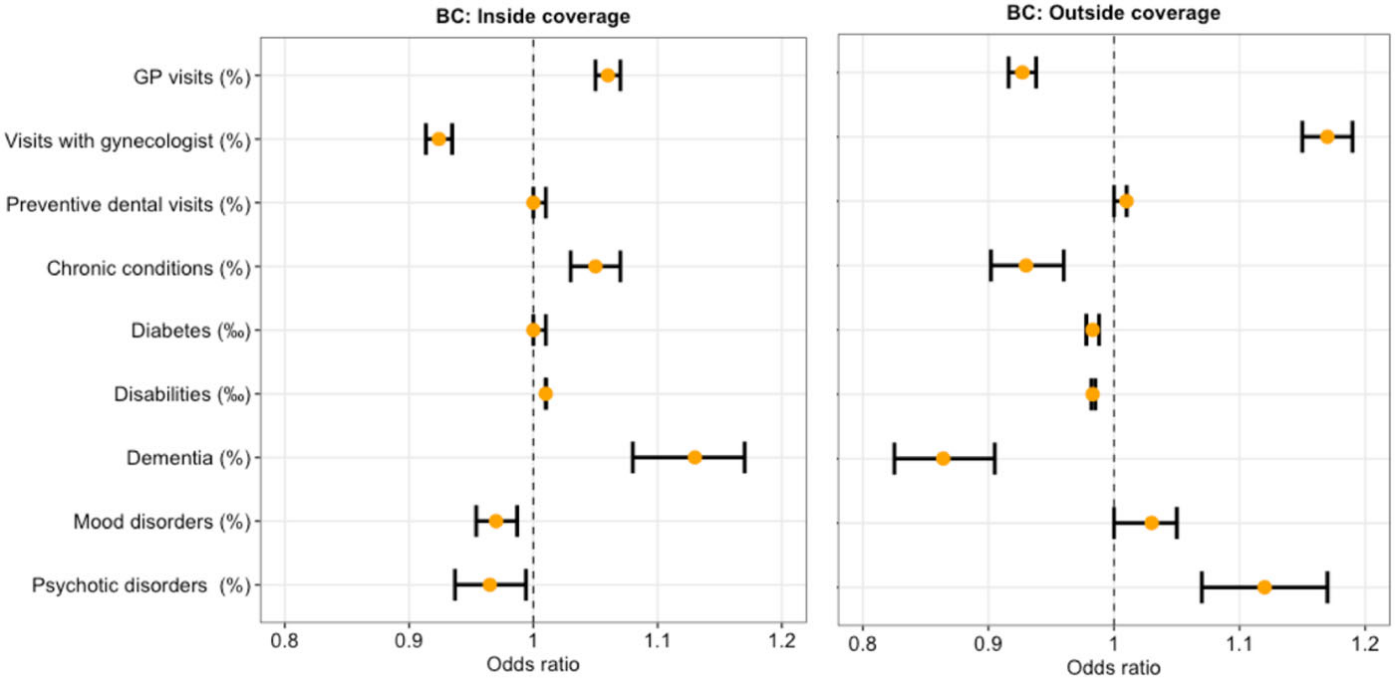
Multivariable association between health-related variables and BC screening

Full results are shown in figure 1 and Supplementary table S4.

### Association between health-related variables and CRC screening

Analogously with BC screening, municipalities with a higher rate of visits with gastroenterologists had 3% lower odds of coverage inside the organized CRC screening program [aOR 0.97 (95% CI 0.96–0.98); *P* < 0.0001]. By contrast, municipalities with a higher rate of GP visits had, respectively, 2% and 5% higher odds of coverage, both inside [aOR 1.02 (95% CI 1.01–1.03); *P* < 0.0001] and outside of the organized program [aOR 1.05 (95% CI 1.04–1.06); *P* < 0.0001].

With respect to health status, municipalities with a higher percentage of people with chronic conditions showed 3% higher odds of CRC screening in the organized screening program [aOR 1.03 (95% CI 1.02–1.04); *P* < 0.0001] and 6% lower odds of coverage outside of the program [aOR 0.94 (95% CI 0.92–0.97); *P* < 0.0001].

Municipalities with a higher percentage of people with dementia also showed 5% higher odds of CRC screening inside the program [aOR 1.05 (95% CI 1.02–1.08); *P* < 0.0001].

Municipalities with a higher percentage of people with mood disorders and alcohol addiction showed, respectively, 4% and 24% lower odds of CRC screening inside of the program [aOR 0.96 (95% CI 0.95–0.97); *P* < 0.0001, and 0.86 (95% CI 0.75–0.99); *P* = 0.03].

The rate of preventive dental visits, percentage of people affected by psychotic disorders, diabetes and with a disability, did not have any significant impact on CRC screening coverage.

**Figure 2:**
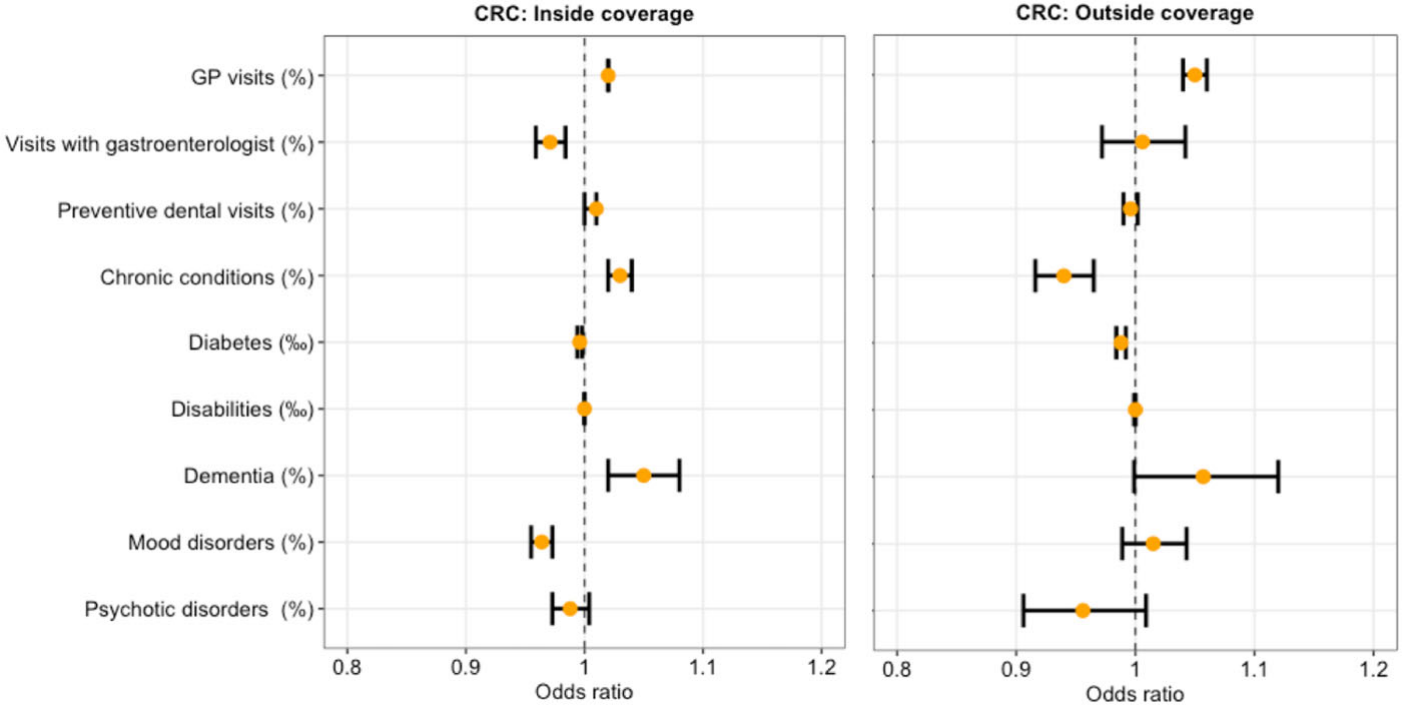
Multivariable association between health-related variables and CRC screening

Full results are shown in figure 2 and Supplementary table S5.

## Conclusion

Our results highlight that physicians play an important role in cancer screening participation choices. Visits to GPs positively influence coverage inside both the BC and CRC program and, in case of FIT testing, also coverage outside of the program. Specialistic visits may have the opposite effect. In fact, with regards to BC screening, a higher number of visits with gynecologists had significantly lower odds of coverage inside of the program and higher odds of coverage outside of the program.

The reason for this association may be that, in order to get a mammogram outside of the program in Flanders, women need a referral letter, which is mostly written by gynecologists and GPs.

Similarly, a higher number of visits with gastroenterologists was associated with lower coverage inside the CRC screening program.

While the relationship between GP visits and higher CRC screening coverage can be explained by the fact that FITs regarded here as ‘opportunistic’ are counted as ‘FITs prescribed by GPs’, the link between visits with gastroenterologists and lower inside coverage is less straightforward. It is reasonable to assume that a number of people who visited the gastroenterologists may have received a colonoscopy or were diagnosed with CRC. As a consequence, some of them did not participate in the program due to an indication for exclusion (not yet registered at the CvKO due to administrative delay).

Belgium represents an interesting scenario to study screening participation patterns. Some of our previous studies have shown a noticeable difference in BC and CRC screening uptake profiles between the northeastern part of Flanders, closer to the border with the Netherlands, and the part of Flanders closer to the French-speaking, southern part of Belgium (Wallonia).^12^^,^^29^ In the latter, most women are screened for BC outside the organized program following consultations with gynecologists or GPs.^30^ Because each municipality in Flanders has relative autonomy in health promotion and disease prevention actions, sociodemographic and cultural differences among these communities (e.g. socio-economic status, number of immigrants, languages) may be responsible for the differential influence that consultations with GPs and specialists have on coverage in and outside of the screening program.

Our results also find support in a vast collection of literature. Recent reviews have demonstrated a clear positive association between provider recommendation and screening adherence.^11^^,^^31^ The role of GPs in influencing screening participation choices was highlighted in previous studies conducted in Flanders at both municipal^12–14^ and individual^32^^,^^33^ levels.

With regards to healthcare access and consumption in general, an analysis of the 2003 World Health Survey has shown that both country health expenditure and healthcare access can influence BC screening.^18^ In a study conducted by Coughlin et al. mammographic screening was positively associated with having had a routine physical examination in the past year, with the number of office-based primary care physicians and the number of health centers or clinics.^17^

A study conducted by Zapka et al. showed that an increased frequency of preventive health visits and ever receiving a physician’s recommendation were associated with higher rates of CRC screening.^16^

With regards to health status, we observed that municipalities with a higher percentage of people with chronic conditions have higher odds of coverage within both the BC and CRC programs and lower odds of coverage outside of the program. In addition, a higher percentage of people with specific health conditions such as diabetes and disabilities was negatively associated with coverage by mammography outside of the organized program. Although other studies conducted in Flanders did not demonstrate a statistically significant association between chronic conditions and screening coverage, specific health conditions such diabetes, a disability or simply older age have been identified as possible barriers to screening for people living in this region^12–14^ In this regard, an analysis from the 2005 US National Health Interview Survey involving over 12 000 individuals showed that, while individuals with a functional limitation or high number of chronic health conditions had higher rates of CRC screening, women reporting poor overall health were less likely to have a mammogram.^18^

We also found that municipalities with a higher percentage of people aged 65+ affected by dementia have higher odds of coverage inside both the BC and CRC screening programs. These findings are in contrast with those of a recent meta-analysis showing that, for women with cognitive impairment or dementia, mammography rates are lower than for those without. The study did not show any significant difference between the rate of CRC screening among individuals with and without dementia.^34^ A possible explanation for these results may be that, as well as for people with other chronic conditions, 65+ affected by dementia would have more frequent interactions with healthcare workers and settings, leading to more frequent screenings. Frequent contacts with healthcare workers can also improve caregivers and family members knowledge and attitude toward preventive care, prompting them to participate in the screening program.

Finally, a higher percentage of people with mood disorders was associated with lower coverage inside of both the BC and CRC screening program. This is in line with a recent meta-analysis indicating that screening was 25% less likely in people with any mental disease. Specifically, people with mood disorders were significantly less likely to get screened for BC but were more likely to get screened for CRC, compared with persons without a mood disorder.^35^

Our results show that municipalities with a higher percentage of people with psychotic disorders have lower odds of coverage inside of the organized BC screening program and higher odds of coverage outside of it. While the first finding aligns with other sources, generally revealing that screening for BC is less likely among people with schizophrenia and other psychotic disorders, who typically experience high rates of poverty and insecure housing,^35^ the latter may be explained by mammograms or referral letters for testing being provided by physicians during outpatient and inpatient visits due to other health concerns. However, further studies will be needed to explain these results.

It should be noted that depression and anxiety have been linked with a significantly increased risk of cancer incidence, cancer-specific mortality and all-cause mortality in cancer patients (+13%, 21% and 24%, respectively)^36^ and that patients with schizophrenia have an approximately 50% increased risk of death by cancer compared with the general population.^37^

Lastly, our results show that municipalities with higher rates of users of anti-alcohol addiction drugs have significantly lower odds of coverage inside of the organized CRC screening program and higher odds of coverage outside of the organized BC screening program. While literature shows that problematic alcohol consumption is associated with lower healthcare use,^38^ higher odds of opportunistic mammography may be attributed to gender differences in its prevalence and patterns. In particular, women who have access to anti-alcohol drugs may belong to higher socioeconomic strata, exhibiting an increased propensity to access paid healthcare services. Additionally, due the well-established association between problematic alcohol use and BC in women, they may have higher likelihood of being referred for BC screening outside of the organized program.

It's important to recognize some limitations of our study. First, the aggregation of data at a municipal level introduces the risk of ecological fallacy.^39^ This potential bias could lead to misinterpretations of individual-level behaviors based on the characteristics of the aggregated group.

Second, our model's independent variables were measured using data encompassing the entire population of each municipality. Their use as a proxy for the study's intended target population introduces the possibility of misalignment with specific demographic under examination.

Thirdly, the assumption that the rate of users of specific drugs reliably represents the prevalence of people psychiatric conditions may not account for variations in diagnosis, treatment and reporting, distorting the associations hypothesized.

Finally, it is important to recognize that testing outside the screening program can happen for both preventive and diagnostic purposes. However, due to the nature of data available, it was not possible to distinguish between outside tests for screening or diagnostic purposes.

In general, the employment of data not specifically designed for addressing our research question may introduce uncertainties in the validity of our findings, warranting caution in generalizing the results to individual-level behaviors.

Despite these limitations, most of our findings can be substantiated with several observations made on a national and international level, supporting their robustness and relevance within broader contexts.

To the best of our knowledge, we were the first to test the associations between a significant number of health-related variables and cancer screening coverage both inside and outside of the organized programs. By applying the Benjamini–Hochberg Procedure, 75% of tests remained statistically significant after correction for multiple testing, indicating the validity of the reported associations.

Our findings suggest that, especially for municipalities characterized by specific health attributes, concerted efforts by the local authorities aimed at actively empower community health providers, including both GPs and specialists, to be involved in their patients’ screening decision-making process, may have the potential to guide the target group toward more optimal cancer screening outcomes.

## Supplementary Material

ckad206_Supplementary_Data

## Data Availability

Data on screening coverage, healthcare utilization and demographic and socioeconomic municipal characteristics in Flanders were retrieved from the Centre for Cancer Detection (CvKO) (https://www.bevolkingsonderzoek.be), the Belgian InterMutualist Agency (IMA) databank (http://atlas.ima-aim.be/databanken) and the Flemish provincial authorities' databank (https://provincies.incijfers.be/databank). Key points Consistently with prior research, municipalities with higher contact with GPs have higher odds of screening inside of the organized program and lower odds of testing outside of the organized program. A similar occurrence applies for municipal characteristics likely to increase healthcare contacts (e.g. chronic conditions, elderly with dementia). Municipalities with higher contact with organ-specific specialists such as gynecologists and gastroenterologists, however, have higher odds of screening outside of the organized program and, for breast cancer, lower odds of screening inside of the organized program. Our findings suggest an opportunity for the local authorities to prioritize the training and proactive engagement of GPs and specialists, as key facilitators for enhancing participation in the organized screening programs.
