# Supplementary material for: Relationship between health-related determinants and adherence to breast and colorectal cancer screening: a population-based study in Flanders, Belgium
Source: Eur J Public Health. 2023 Nov 24;34(2):347–53. doi: 10.1093/eurpub/ckad206 (PMC10990537; doi:10.1093/eurpub/ckad206)
Supplement: ckad206_Supplementary_Data [file ckad206_supplementary_data.docx]

**Supplementary materials**

**Figure S1 - Causal directed acyclic graph (DAG) for the analysis of causal inference (BC screening)**

Available at: [dagitty.net/mkiMrdu](http://dagitty.net/mkiMrdu).

**
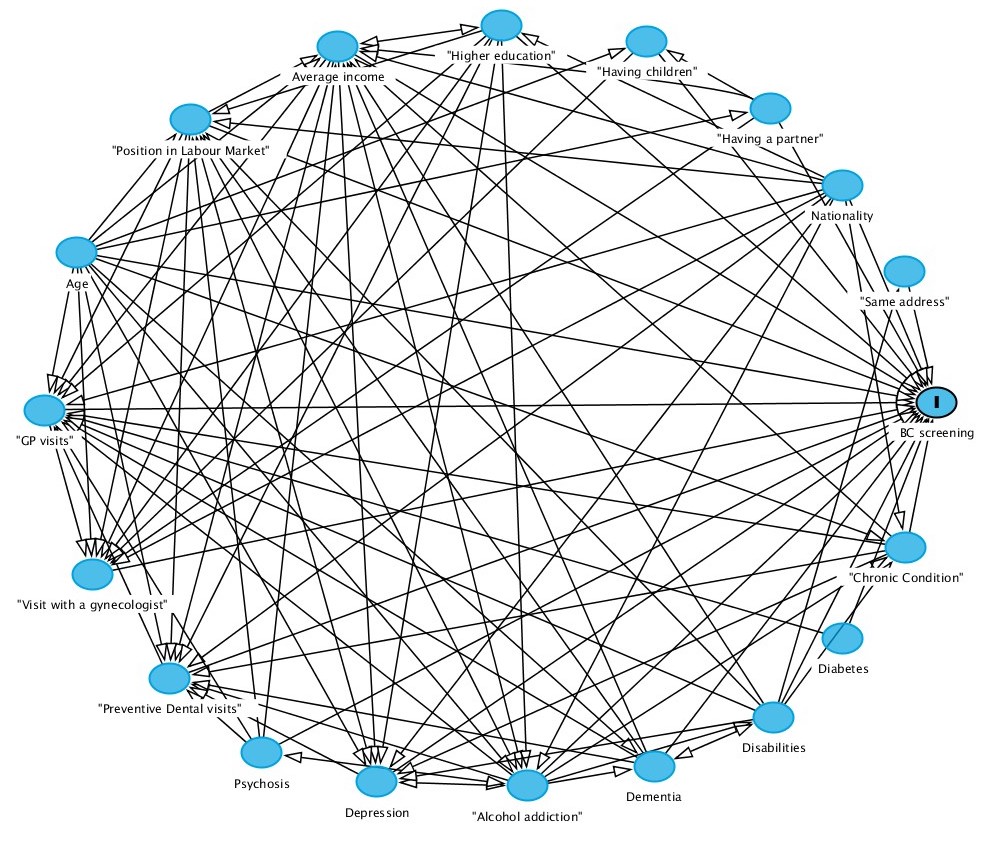
**

**Table S1 - List of covariates for adjustment in multivariable analyses to estimate the association between main health-related variables and BC screening coverage, based on DAG**

| **Main determinant of Assessment** | **Covariates for Adjustment in Multivariable Analysis** |
| --- | --- |
| GP visits (%) | Higher education, Position in labor market, Nationality, Age group, Average income, Preventive Dental visits, Chronic Conditions, People with diabetes, People with disabilities, People with dementia, People with psychotic disorders, People with alcohol addiction |
| Visits with gastroenterologist (%) | People with a partner, People with children, Higher education, Position in labor market, Age group, Average income, Nationality, GP visits |
| Preventive dental visits (%) | Higher education, Position in labor market, Nationality, Age group, Average income, Chronic Conditions, People with dementia, People with psychotic disorders, People with alcohol addiction |
| Chronic conditions (%) | Age group, Nationality, People with disabilities, People with alcohol addiction |
| Diabetes (‰) | / |
| Disabilities (‰) | Age group, People with dementia, People with alcohol addiction |
| Dementia (%) | Age group, People with disabilities, People with alcohol addiction |
| Mood disorders (%) | Higher education, Position in labor market, Nationality, Age group, People with a partner, Average income, Chronic Conditions, People with disabilities, People with dementia, People with psychotic disorders, People with alcohol addiction |
| Psychotic disorders (%) | Higher education, Position in labor market, Nationality, Age group, People with a partner, Average income, GP visits, Preventive dental visits, Chronic Conditions, People with diabetes, People with disabilities, People with dementia, People with mood disorders, People with alcohol addiction (direct effect only*) |
| Alcohol additction (%) | Higher education, Position in labor market, Nationality, Age group, People with a partner, Average income, GP visits, Chronic Conditions, Preventive dental visits, People with diabetes, People with disabilities, People with dementia, People with mood disorders, People with psychotic disorders (direct effect only*) |

**Figure S2 - Causal directed acyclic graph (DAG) for the analysis of causal inference (CRC screening)**

Available at: [dagitty.net/mOMmJE3](http://dagitty.net/mOMmJE3)

**
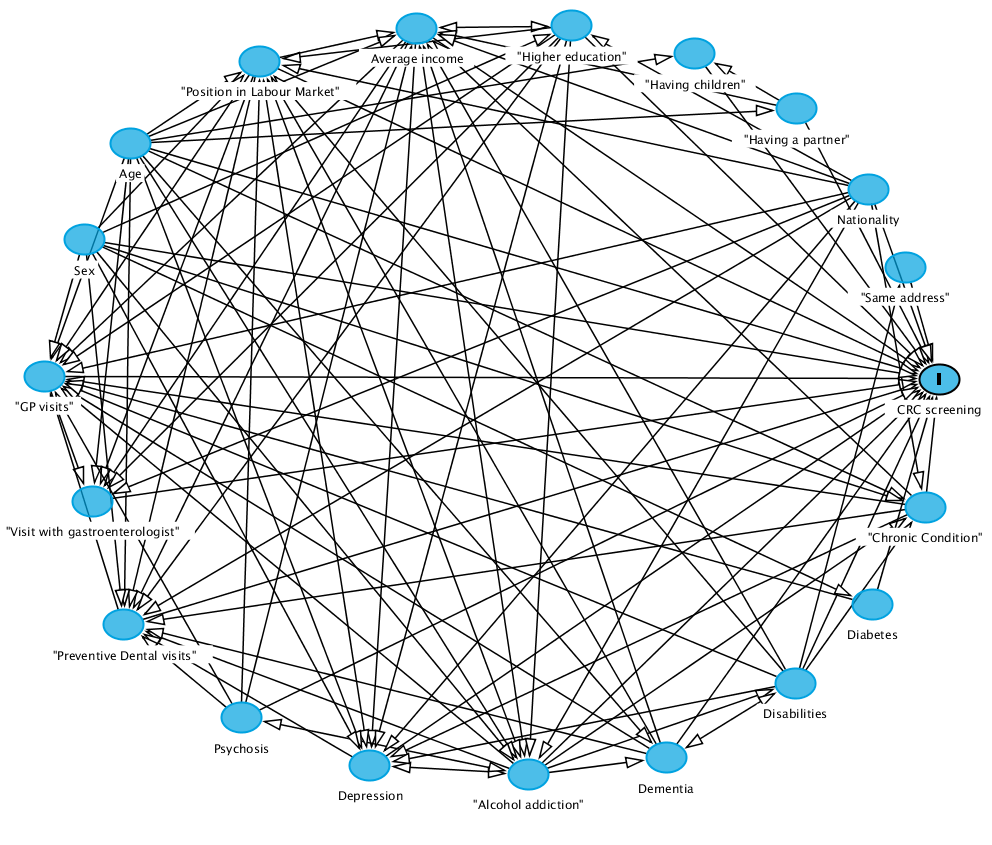
**

**Table S2 - List of covariates for adjustment in multivariable analyses to estimate the association between main health-related variables and CRC screening coverage, based on DAG**

| **Main determinant of Assessment** | **Covariates for Adjustment in Multivariable Analysis** |
| --- | --- |
| GP visits (%) | Higher education, Position in labor market, Nationality, Age group, Sex, Average income, Preventive Dental visits, Chronic Conditions, People with diabetes, People with disabilities, People with dementia, People with psychotic disorders, People with alcohol addiction |
| Visits with gastroenterologist (%) | Higher education, Position in labor market, Age group, Average income, Nationality, GP visits |
| Preventive dental visits (%) | Higher education, Position in labor market, Nationality, Age group, Sex, Average income, Chronic Conditions, People with dementia, People with psychotic disorders, People with alcohol addiction |
| Chronic conditions (%) | Age group, Nationality, People with disabilities, People with alcohol addiction |
| Diabetes (‰) | Sex |
| Disabilities (‰) | Age group, People with dementia, People with alcohol addiction |
| Dementia (%) | Age group, People with disabilities, People with alcohol addiction |
| Mood disorders (%) | Higher education, Position in labor market, Nationality, Age group, Sex, People with a partner, Average income, Chronic Conditions, People with disabilities, People with dementia, People with psychotic disorders, People with alcohol addiction |
| Psychotic disorders (%) | Higher education, Position in labor market, Nationality, Age group, Sex, People with a partner, Average income, GP visits, Preventive dental visits, Chronic Conditions, People with diabetes, People with disabilities, People with dementia, People with mood disorders, People with alcohol addiction (direct effect only*) |
| Alcohol additction (%) | Higher education, Position in labor market, Nationality, Age group, Sex, People with a partner, Average income, GP visits, Preventive dental visits, Chronic Conditions, People with diabetes, People with disabilities, People with dementia, People with mood disorders, People with psychotic disorders (direct effect only*) |

**Table S3 – Breast (BC) and colorectal cancer (CRC) screening coverage, inside and outside the organized program in Flanders (2015-2018)**; Median (25^th^-75^th^ percentiles)

| **Year** | **BC screening inside the program** | **BC screening outside the program** | **CRC screening inside the program** | **CRC screening outside the program )** |
| --- | --- | --- | --- | --- |
| 2015 | 50.00 (43.50-54.90) | 51.30 (44.60-55.80) | 51.80(45.10-55.90) | 51.70 (44.60-55.70) |
| 2016 | 12.20 (9.07-17.80) | 11.90 (8.80-17.60) | 12.00 (8.58-17.10) | 11.50 (7.88-17.00) |
| 2017 | 36.30 (33.60-39.20) | 37.90 (35.10-40.05) | 39.90 (37.2-42.8) | 36.40 (33.70-39.00) |
| 2018 | 4.80 (3.60-6.30) | 3.90 (3.00-5.00) | 3.30 (2.60-4.30) | 3.10 (2.48-4.00) |

**Table S4 – Multivariable association between health-related variables and BC screening**

| **Health-related variables** | **Coverage inside the BC screening program** | | | | **Coverage outside the BC screening program** | | | |
| --- | --- | --- | --- | --- | --- | --- | --- | --- |
|  | **OR** | **95%L** | **95%H** | **p value** | **OR** | **95%L** | **95%H** | **p value** |
| GP visits (%) | 1.06 | 1.05 | 1.07 | <0.0001* | 0.93 | 0.92 | 0.94 | <0.0001* |
| Visits with gynecologist (%) | 0.92 | 0.91 | 0.93 | <0.0001* | 1.17 | 1.15 | 1.19 | <0.0001* |
| Preventive dental visits (%) | 1.00 | 1.00 | 1.01 | 0.0589 | 1.01 | 1.00 | 1.01 | 0.0065 |
| Chronic conditions (%) | 1.05 | 1.03 | 1.07 | <0.0001* | 0.93 | 0.90 | 0.96 | <0.0001* |
| Diabetes (‰) | 1.00 | 1.00 | 1.01 | 0.0152 | 0.98 | 0.98 | 0.99 | <0.0001* |
| Disabilities (‰) | 1.01 | 1.00 | 1.01 | <0.0001 | 0.98 | 0.98 | 0.99 | <0.0001* |
| Dementia (%) | 1.13 | 1.08 | 1.17 | <0.0001* | 0.86 | 0.83 | 0.91 | <0.0001* |
| Mood disorders (%) | 0.97 | 0.95 | 0.99 | 0.0004* | 1.03 | 1.00 | 1.05 | 0.0255 |
| Psychotic disorders (%) | 0.97 | 0.94 | 0.99 | 0.0166* | 1.12 | 1.07 | 1.17 | <0.0001* |
| Alcohol additction (%) | 1.06 | 0.83 | 1.37 | 0.64 | 1.65 | 1.12 | 2.44 | 0.0119 |

**Table S5 – Multivariable association between health-related variables and CRC screening**

| **Health-related variables** | **Coverage inside the CRC screening program** | | | | **Coverage outside the CRC screening program** | | | |
| --- | --- | --- | --- | --- | --- | --- | --- | --- |
|  | **OR** | **95%L** | **95%H** | **p value** | **OR** | **95%L** | **95%H** | **p value** |
| GP visits (%) | 1.02 | 1.01 | 1.03 | <0.001* | 1.05 | 1.03 | 1.08 | <0.001* |
| Visits with gastroenterologist (%) | 0.97 | 0.95 | 0.99 | 0.02* | 1.01 | 0.95 | 1.07 | 0.84 |
| Preventive dental visits (%) | 1.0061 | 1.0023 | 1.0098 | <0.01* | 0.9958 | 0.9870 | 1.0040 | 0.35 |
| Chronic conditions (%) | 1.03 | 1.01 | 1.05 | <0.001* | 0.94 | 0.90 | 0.98 | < 0.01* |
| Diabetes (‰) | 0.9965 | 0.9942 | 0.9967 | <0.01* | 0.9888 | 0.9832 | 0.9943 | <0.001* |
| Disabilities (‰) | 1.0023 | 1.0013 | 1.0031 | <0.001* | 1.0003 | 0.9980 | 1.0025 | 0.79 |
| Dementia (%) | 1.05 | 1.02 | 1.08 | <0.001* | 1.06 | 0.99 | 1.13 | 0.12 |
| Mood disorders (%) | 1.06 | 0.99 | 1.13 | 0.12 | 1.02 | 0.97 | 1.07 | 0.55 |
| Psychotic disorders (%) | 0.96 | 0.94 | 0.98 | <0.001* | 0.96 | 0.87 | 1.05 | 0.34 |
| Alcohol additction (%) | 0.99 | 0.95 | 1.03 | 0.54 | 0.66 | 0.30 | 1.46 | 0.31 |

| **Outcome** | **Health-related variables** | **OR** | **L 95%** | **H 95%** | **P value** | **Rank (i)** | **(i/m)*Q** |
| --- | --- | --- | --- | --- | --- | --- | --- |
| BC Inside coverage | Gynecologist visits (%) | 0.9240 | 0.9135 | 0.9346 | <0.001 | 1 | 0.001 |
| BC Inside coverage | GP visits (%) | 1.0600 | 1.0500 | 1.0700 | <0.001 | 2 | 0.003 |
| BC Inside coverage | Chronic conditions (%) | 1.0500 | 1.0300 | 1.0700 | <0.001 | 3 | 0.004 |
| BC Inside coverage | Disabilities (‰) | 1.0100 | 1.0100 | 1.0100 | <0.001 | 4 | 0.005 |
| BC Inside coverage | Dementia (%) | 1.1300 | 1.0800 | 1.1700 | <0.001 | 5 | 0.006 |
| BC Outside coverage | Gynecologist visits (%) | 1.1700 | 1.1500 | 1.1900 | <0.001 | 6 | 0.008 |
| BC Outside coverage | GP visits (%) | 0.9270 | 0.9160 | 0.9380 | <0.001 | 7 | 0.009 |
| BC Outside coverage | Chronic conditions (%) | 0.9300 | 0.9020 | 0.9600 | <0.001 | 8 | 0.010 |
| BC Outside coverage | Diabetes (‰) | 0.9830 | 0.9780 | 0.9880 | <0.001 | 9 | 0.011 |
| BC Outside coverage | Disabilitiesx(‰) | 0.9830 | 0.9820 | 0.9850 | <0.001 | 10 | 0.013 |
| BC Outside coverage | Psycothic disorders (%) | 1.1200 | 1.0700 | 1.1700 | <0.001 | 11 | 0.014 |
| BC Outside coverage | Dementia (%) | 0.8640 | 0.8250 | 0.9050 | <0.001 | 12 | 0.015 |
| CRC Inside coverage | Gastroeneterologist visits (%) | 0.9710 | 0.9590 | 0.9840 | <0.001 | 13 | 0.016 |
| CRC Inside coverage | GP visits (%) | 1.0200 | 1.0200 | 1.0200 | <0.001 | 14 | 0.018 |
| CRC Inside coverage | Preventive oral care visits (%) | 1.0100 | 1.0000 | 1.0100 | <0.001 | 15 | 0.019 |
| CRC Inside coverage | Chronic conditions (%) | 1.0300 | 1.0200 | 1.0400 | <0.001 | 16 | 0.020 |
| CRC Inside coverage | Disabilities (‰) | 1.0000 | 1.0000 | 1.0000 | <0.001 | 17 | 0.021 |
| CRC Inside coverage | Mood disorders (%) | 0.9640 | 0.9550 | 0.9730 | <0.001 | 18 | 0.023 |
| CRC Outside coverage | GP visits (%) | 1.0500 | 1.0400 | 1.0600 | <0.001 | 19 | 0.024 |
| CRC Outside coverage | Chronic conditions (%) | 0.9400 | 0.9160 | 0.9650 | <0.001 | 20 | 0.025 |
| CRC Outside coverage | Diabetes (‰) | 0.9880 | 0.9840 | 0.9920 | <0.001 | 21 | 0.026 |
| CRC Inside coverage | Dementia (%) | 1.0500 | 1.0200 | 1.0800 | 0.0002 | 22 | 0.028 |
| CRC Inside coverage | Diabetes (‰) | 0.9960 | 0.9940 | 0.9980 | 0.0003 | 23 | 0.029 |
| BC Inside coverage | Mood disorders (%) | 0.9700 | 0.9540 | 0.9870 | 0.0004 | 24 | 0.030 |
| BC Outside coverage | Preventive oral care visits (%) | 1.0100 | 1.0000 | 1.0100 | 0.0065 | 25 | 0.031 |
| BC Outside coverage | Alcohol addiction (%) | 1.6500 | 1.1200 | 2.4400 | 0.0119 | 26 | 0.033 |
| BC Inside coverage | Diabetesx1000 (%) | 1.0000 | 1.0000 | 1.0100 | 0.0152 | 27 | 0.034 |
| BC Inside coverage | Psycothic disorders (%) | 0.9650 | 0.9370 | 0.9940 | 0.0167 | 28 | 0.035 |
| BC Outside coverage | Mood disorders (%) | 1.0300 | 1.0000 | 1.0500 | 0.0255 | 29 | 0.036 |
| CRC Inside coverage | Alcohol addiction (%) | 0.8560 | 0.7450 | 0.9850 | 0.0298 | 30 | 0.038 |
| CRC Outside coverage | Dementia (%) | 1.0570 | 0.9990 | 1.1200 | 0.0549 | 31 | 0.039 |
| BC Inside coverage | Preventive oral care visits (%) | 1.0000 | 1.0000 | 1.0100 | 0.0589 | 32 | 0.040 |
| CRC Outside coverage | Alcohol addiction (%) | 0.6610 | 0.4270 | 1.0020 | 0.0627 | 33 | 0.041 |
| CRC Outside coverage | Psycothic disorders (%) | 0.9560 | 0.9060 | 1.0090 | 0.1023 | 34 | 0.043 |
| CRC Inside coverage | Psycothic disorders (%) | 0.9880 | 0.9730 | 1.0040 | 0.1410 | 35 | 0.044 |
| CRC Outside coverage | Preventive oral care visits (%) | 0.9960 | 0.9900 | 1.0020 | 0.1527 | 36 | 0.045 |
| CRC Outside coverage | Mood disorders (%) | 1.0150 | 0.9890 | 1.0430 | 0.2664 | 37 | 0.046 |
| BC Inside coverage | Alcohol addiction (%) | 1.0610 | 0.8250 | 1.3660 | 0.6430 | 38 | 0.048 |
| CRC Outside coverage | Disabilities (‰) | 1.0000 | 0.9990 | 1.0002 | 0.7148 | 39 | 0.049 |
| CRC Outside coverage | Gastroeneterologist visits (%) | 1.0060 | 0.9720 | 1.0420 | 0.7214 | 40 | 0.050 |

**Table S6 - Logistic Regression results adjusted by Benjamini-Hochberg Procedure to control for multiple testing**
